# Study on reasonable anchorage length based on failure mechanism of the bolt anchorage system

**DOI:** 10.1038/s41598-023-45778-w

**Published:** 2023-12-21

**Authors:** Meng Wang, Liyou Shang, Baoan Zhang, Yatao Li, Jinshuai Su, Shuai Wang

**Affiliations:** College of Mining, Liaoning Technical University, Fuxin, Liaoning China

**Keywords:** Engineering, Civil engineering

## Abstract

In addition to analysing the mechanism of failure of the prestressed rock anchor anchor system and investigating the appropriate depth for fixing the rock anchors, theoretical equations were derived to calculate the rock anchors' axial force, ultimate capacity, and the interfacial shear force in the elastic phase. These equations are then used to analyse the pressure distribution within the rock bolt anchorage section and to investigate the effect of interfacial shear strength, shear stiffness, and anchorage length on interface failure. Drawing on the findings from both field-based rock bolt pull-out tests and numerical simulations, analyzed the failure mechanism of the anchor system, and proposed a reasonable anchor length design method for rock bolt. The results show that there is a strong dependence between ultimate load carrying capability of rock bolts and interfacial shear stress and shear rigidity, and that increasing the anchorage length and reducing the interface shear stiffness can avoid the stress concentration phenomenon. The primary factor leading to the anchor system failure is secondary interface failure. The evolution law of interface damage is that the damage occurs first at the initial position. As the interface damage location changes, the peak shearing stress moves towards the bottom of the anchored section. The engineering application results verified the feasibility of a reasonable anchorage length calculation method and rock bolt design process. The findings of this paper can be used as a basic reference for determining rock bolt anchorage support parameters during the design and construction of underground engineering projects.

## Introduction

Resin anchoring technology is widely used in water conservancy and hydropower, mine shaft, tunnel, and slope reinforcement projects^1^. The fundamental principle of resin anchoring technology is to enhance the stability of the reinforced structure by securely bonding the rock bolt to the geotechnical body using the adhesive properties of the resin anchoring agent^2–4^. The combined factors of the rock conditions, the bonding properties of the resin rolls as well as the construction process can create a stressful environment. This often leads to rock bolt slip damage, resulting in severe deformation of the pavement perimeter rock^5,6^. Anchorage length is an essential factor affecting the tensile performance and load-carrying capacity of rock bolt. Factors affecting anchorage length include geological conditions, anchor diameter, anchor material, anchorage method, etc. Different factors require different anchorage lengths. Different factors have different requirements for anchorage length, so the reasonable anchorage length must be determined by considering all factors according to the actual situation^7^.

Therefore, clarifying the mechanism of failure of the rock body bolt anchorage in resin rock bolt reinforcement works and exploring the reasonable anchorage length of rock bolts have been one of the key issues in this research field^8^. Hobst^9^ pointed out that anchorage length is a significant factor that affects the anchorage capacity of prestressing anchors. Pang et al.^10^ derived the mechanical formulae for the calculation of axial force and the shear stress of the anchors by establishing a mechanical model for the interaction between full-length rock anchors and the surrounding rock. Wang et al.^11^ conducted a comprehensive study on the effects of anchor rod depth and preload force on the effectiveness of rock control. To achieve this, they carried out theoretical analyses and field tests to investigate the stress pattern distribution in the surrounding stone under different anchor lengths and preload conditions. Zhao et al.^12^ developed a comprehensive anchor-rock interaction model which takes into account the deformation behaviour in the enclosing rock. According to this mould, they derived expressions for the analytical analysis of axial and shear stresses in the fully anchored anchorage under normal support conditions as well as in the case of critical damage. Richard et al.^13^ found through field tests that there is a phenomenon of shear slip between the anchorage body and the prestressing anchor during the process of anchor pull-out. Ehsani et al.^14^ derived the procedure for calculating the anchor anchorage length under varying surrounding rock parameters through an examination of diverse adhesive shear-slip curve models at different anchor anchorage interfaces. Evangelista et al.^15^ observed the phenomenon of critical anchorage length of anchors in hard sandy soil, non-cohesive soil, and loess soil, respectively. Zhao et al.^12^ investigated the critical anchorage length of BFRP (Basalt fiber reinforced polymer) cement mortar anchors using a combination of numerical simulations and indoor tests. Xu et al.^16^ investigated the effect of anchorage length on bond strength through experimental tests. In addition, they explored the effect of protection layer depth on anchorage length with theoretical analyses, and subsequently derived the commonly used formulae for the anchorage length of reinforcing bars in building structures. The above findings provide valuable insights into failure mechanisms in rock bolted anchorage systems and the determination of appropriate anchorage lengths. However, it is important to note that the resin anchorage system in coal tunnels exhibits various failure modes, which may require further investigation and consideration, it is more meaningful to carry out research on rock bolt bearing capacity and reasonable anchor length in combination with failure modes for engineering guidance^17,18^. Therefore, in this study, theoretical analyses, field tests and numerical simulations are used to investigate the characteristics of stress distribution in rock anchors and the mechanical transfer mechanism between force-transmitting media in the anchoring system. The aim is to determine appropriate lengths for anchor sections, which is essential for guiding engineering practice, optimizing rock bolt anchor length designs and enhancing the quality of roadway support.

This paper comprises the following sections: In the first part, the introduction is presented. The second part of the study analyses the stress distributing law of the anchoring system. Part third examines the factors that influence anchorage failure. The fourth part presents the field test and numerical simulation results. The fifth part analyzes appropriate bolt anchorage lengths. Finally, in the sixth part, conclusions are drawn based on the research findings.

## Analysis of stress distribution in anchorage system

### Analytical calculation of stress distribution in rock bolt

Due to the existence of multiple definitions for partial concepts in the literature, it is necessary to clarify the concept first^19–21^. For the purposes of this paper, an anchoring system is defined as a whole consisting of a stone bolt, an anchoring agent, a portion of the surrounding stone body being anchored, and two interfaces between the three. Anchor is a monolithic assembly of rock bolts and anchors. In context, first interface refers to the contact surface of the rock bolt body with the anchoring agent, while the second refers to the interface between the anchored solid and the rock surrounded by the anchored rock.

**Figure 1:**
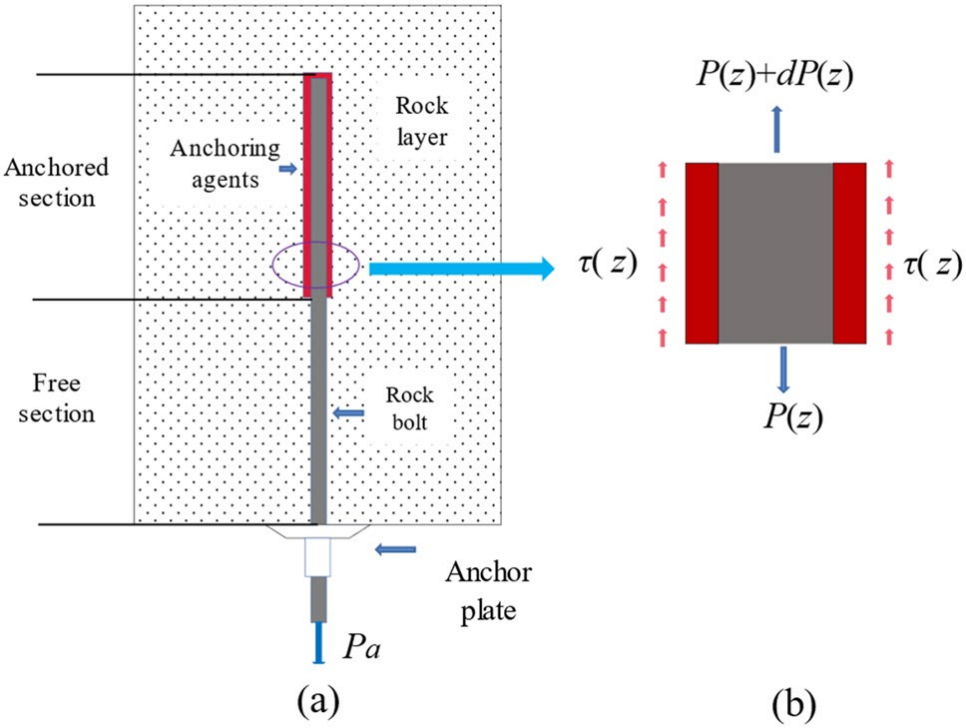
Diagram of single rock bolt anchorage system.

Improvement of the effectiveness of anchor prestressing in reinforcing the roadway perimeter rock surface, traditional practices for coal mine support usually use non-full-length binder anchoring techniques. Figure 1a depicts the modelling of the bolted rock anchorage system. Deformation characteristics of a composite consisting of rock bolts and resin anchors follow Hooke's law. The anchoring length of the rock anchor is La, the diameter of the borehole is D, the danger of the rock anchor is noted as d, and it is subjected to a preload force of Pa. A micro-anchor unit with length *d*z is taken at the anchor section z. As shown in Fig. 1b, the anchor stress at the anchor-grouted rock interface is denoted as *τ*(z), and the elongation of the rock bolt is denoted as s(z).

According to the mechanical equilibrium of the microcellular body, the relations between the thrust force *P*(z) and the deflection *s*(z) of the bolt are:

1 $$ \frac{dP(z)}{{dz}} = \pi D\tau (z) $$

2 $$ \frac{ds(z)}{{dz}} = \frac{4P(z)}{{\pi D^{2} E}} $$

3 $$ E = \frac{{E_{a} (D^{2} - d^{2} ) + E_{b} d^{2} }}{{D^{2} }} $$

which *E* is the elastic complex modulus of the rocks bolt and anchorage; *E*_*a*_ is the elastic modulus of the anchorage; *E*_*b*_ is the elastic modulus of the rocks bolt.

According to a three-stage function for interfacial shear stress-displacement relationship proposed by Benmochlen et al.^22^, the shear pressure distributed at the interface of anchor and surrounding stone can be expressed as:

4 $$ \tau (z) = Ks(z) $$

where *K* is the shear stiffness of the interface.

Combining the equations above, we arrive at Eq. (5).

5 $$ \frac{{d^{2} s(z)}}{{dz^{2} }} - \beta^{2} s(z) = 0 $$

where *β*^2^ = 4* K*/*ED*.

The general solution of the differential Eq. (5) is:

6 $$ s(z) = C_{1} \cosh (\beta z) + C_{2} \sinh (\beta z) $$

where C_1_ and C_2_ are constants of integration in the general solution.

Through the boundary conditions *P*(*z*)|_*z*=0_ = *P*_*a*_ and *P*(*z*)|_*z*=*La*_ = 0, the expressions of axial force in the anchorage section and shear stress distribution at the anchorage-envelope interface are formulas (7) and (8) respectively.

7 $$ P(z) = P_{a} \frac{{\sinh [\beta (L_{a} - z)]}}{{\sinh (\beta L_{a} )}} $$

8 $$ \tau (z) = \frac{{\beta P_{a} \cosh [\beta (L_{a} - z)]}}{{\pi D\sinh (\beta L_{a} )}} $$

Let *τ*(z) be the shear strength [*τ*] at the interface between the anchor and the perimeter stone. Further deduced from formula (7) yields the rock bolt's elastic ultimate bearing capacity Pe, as shown in formula (9).

9 $$ P_{e} = \frac{\pi D[\tau ]}{\beta }\tanh (\beta L_{a} ) $$

### Stress distribution rule along anchorage length

**Figure 2:**
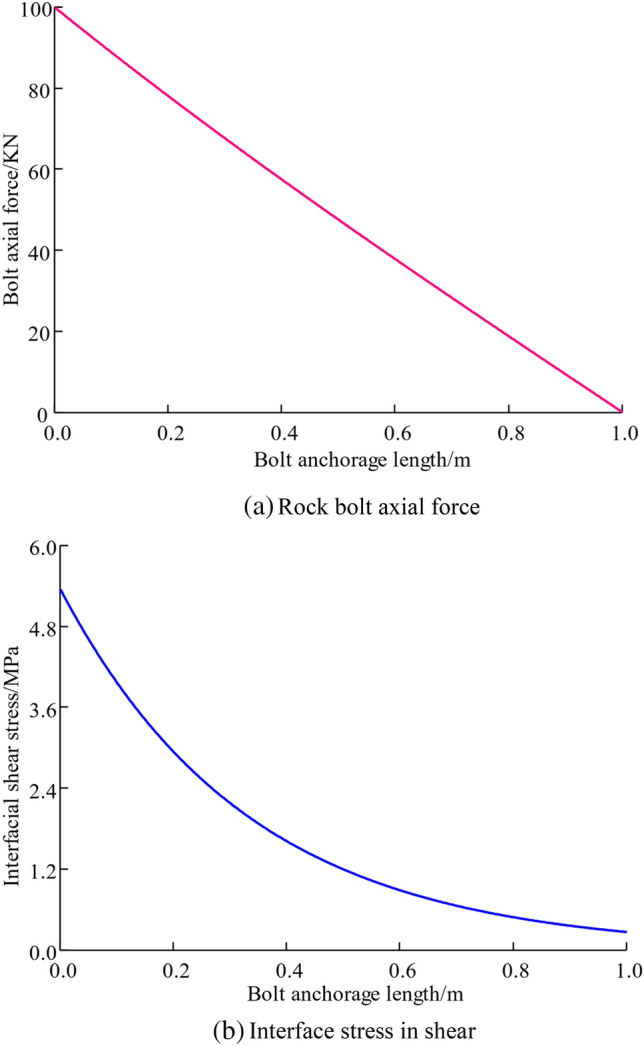
Assignment of rock bolt axial force and interfacial shear stresses.

Investigation of axial anchor stress and anchorage-surrounding rock interface shear stress distribution patterns along the length of the anchorage in the anchored section, Elastic modulus of rock bolt *E*_*b*_ was selected as 2 × 10^5^ MPa, with a diameter of 22 mm. The anchorage has a modulus of elasticity *E*_*a*_ of 1.6 × 10^4^ MPa, and the thickness of the anchoring agent ring is 4 mm. The conjunction with the actual project to choose shear rigidity of the interface is *K* = 300 MPa/m. For an anchorage length of 1 m and pre-tightening force *P*_*a*_ = 100kN, the Fig. 2 shows the axis force distribution in the anchored section as well as the shear force distribution at interface between the anchorage and the surrounding stone.

It can be seen from Fig. 2a shows a linear decrease in rock bolt axial force along the anchorage depth direction, while Fig. 2b indicates that interface shear stress is highest at the start of the anchorage section and gradually stabilizes as depth increases, indicating that the further away from the beginning of the anchorage, the lower the ability of the interface shear stress to provide pullout resistance.

## Anchorage system failure form and influence factors

### Forms of anchoring system failure

Failure of the anchoring system under the effect of preload takes three main forms, excluding failure of rock bolts and anchors due to insufficient strength due to damage.

**Figure 3:**
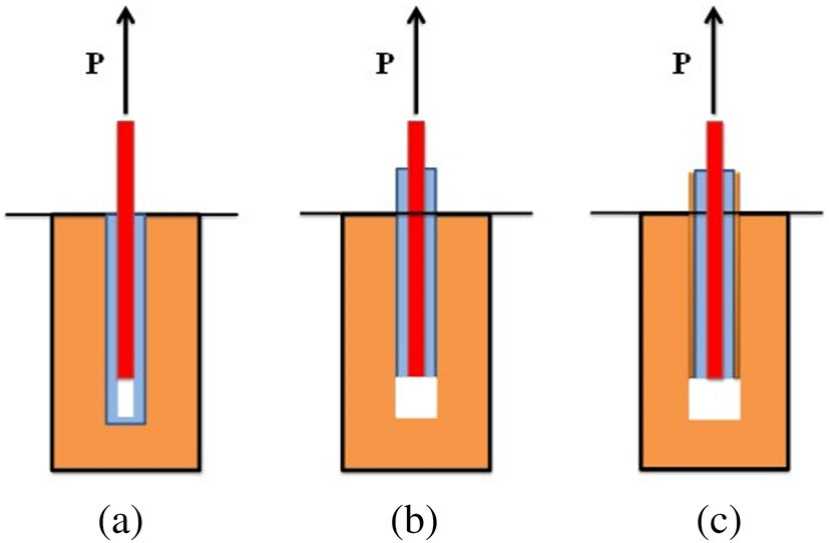
Failure modes of anchoring systems.

1. The first interface failure. The stone bolt was completely pulled out of the anchorage as shown in Fig. 3a.
2. The second interface damage occurred between anchors and the surround rock around drill hole, the whole anchors were detached from the drill hole, as shown in Fig. 3b.
3. The whole of the anchor solid and part of the surrounding rock is pulled out, and the damaged surface enters the surrounding rock by a few millimetres, which usually occurs in the fragile fractured perimeter stone, as shown in Fig. 3c.

### Analysis of factors affecting the ultimate load carrying capability of bolts

#### Interfacial shear strength [*τ*]

**Figure 4:**
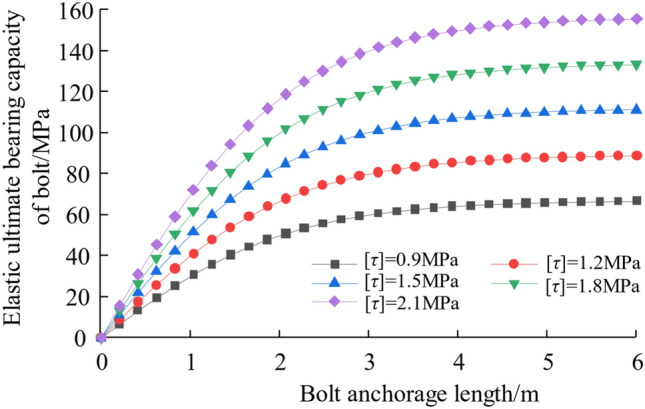
Relationships between ultimate pull-out capacity and anchorage length.

Taking interfacial shell rigidity *K* = 300 MPa/m, interfacial shearing intensity [*τ*] = 0.9 ~ 2.1 MPa. And combine with Eq. (9) to get the relationship curve of rock bolt elastic ultimate bearing capacity under different interface shear strengths with the change of corky anchorage length, as shown below in Fig. 4. As can be seen from the figure, the higher the interfacial shedding strength, the higher the rock bolt elastic ultimate bearing capacity. Increasing the anchorage length and interface shear strength can improve the bolt's ultimate bearing capacity.

#### The influence of interfacial shelf rigidity *K*

**Figure 5:**
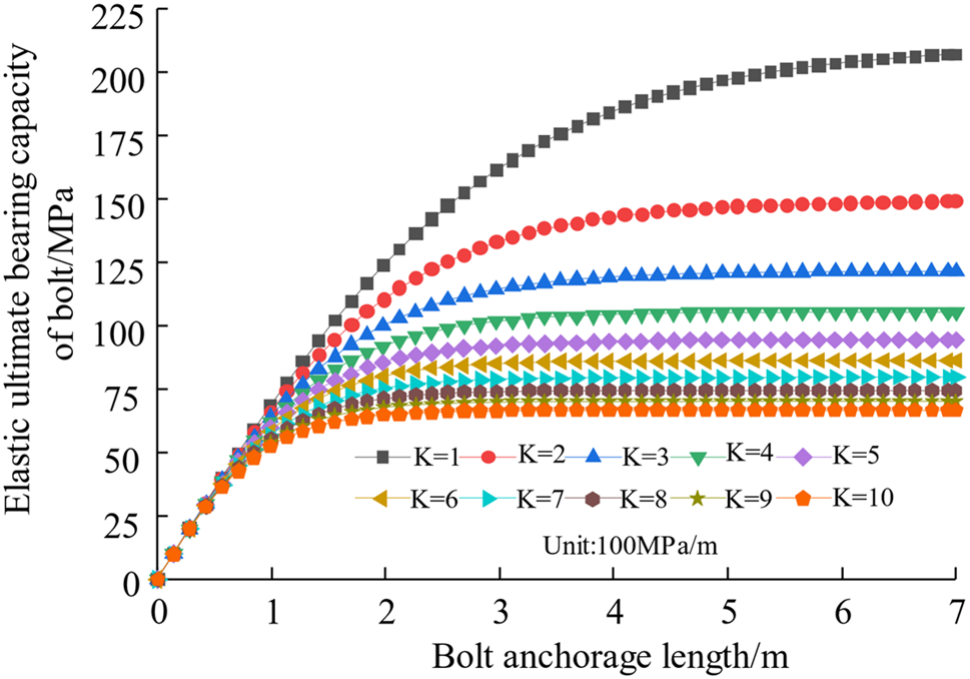
Relationship between different interface shear stiffness and ultimate pullout force of the bolt.

Take the interfacial cut intensity [*τ*] to 1.5 MPa and the interface shear stiffness *K* to 100–1000 MPa/m. In Fig. 5, the curve illustrates the dependence of the elastic ultimate capacity of the rock bolt on the anchorage length. This relationship is examined under varying interface shear stiffness conditions, combined with Eq. (8). Figure 5 shows that the rock bolt's ultimate bearing capacity has a non-linear inverse relationship with the interface shear stiffness. Increasing the anchorage length and reducing the interface shear stiffness can improve the rock bolt's elastic ultimate bearing capacity.

### Analysis of factors influencing interface shear stress

#### Influence of bolt anchor length

**Figure 6:**
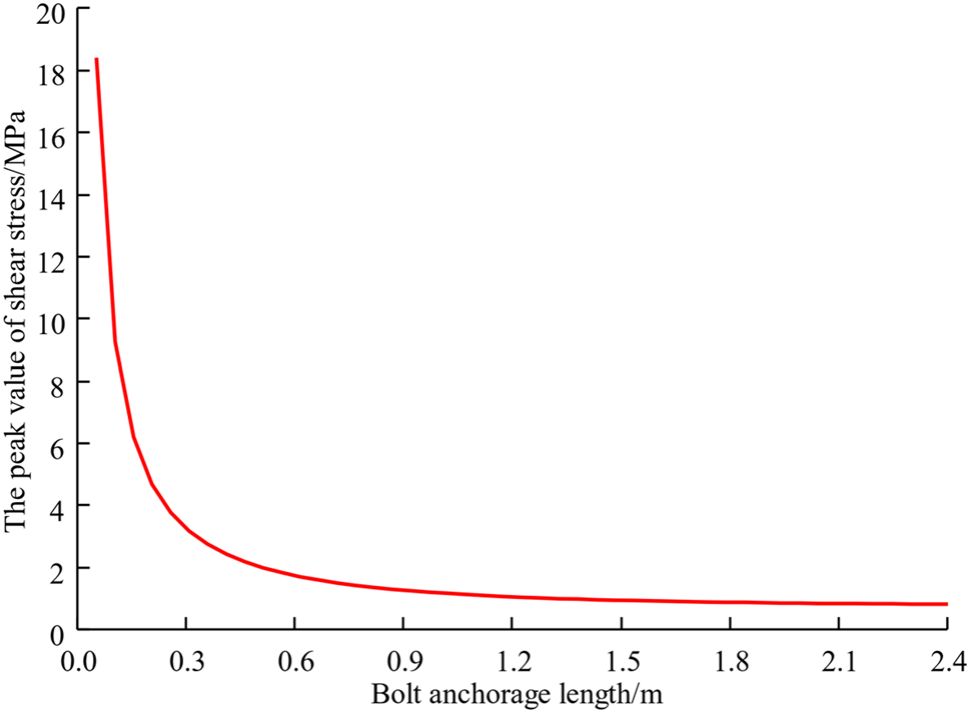
The peak shear stress of different anchorage length.

The magnitude of the shear stress at the anchorage interface is an essential factor affecting whether or not the anchorage system is damaged. According to the previous analysis, the peak value of interfacial shedding stresses usually occurs near the initiation points in anchor segments. Combining with Eq. (8), taking the bolt preload force *P*_*a*_ = 100kN and interfacial shear stiffness *K* = 500 MPa/m, in Fig. 6. The change curve of the peak of interfacial shear stress is depicted for various installation depths. As can be seen from the graph, it is evident that when the anchorage length is less than 0.3 m, peak shearing stress at the interfaces is much higher. The peak shear stress, however, decreases at a faster rate as the anchorage length increases. Therefore, in practical engineering, the anchor length should be greater than 0.3 m.

#### The influence of interfacial shear stiffness *K*

**Figure 7:**
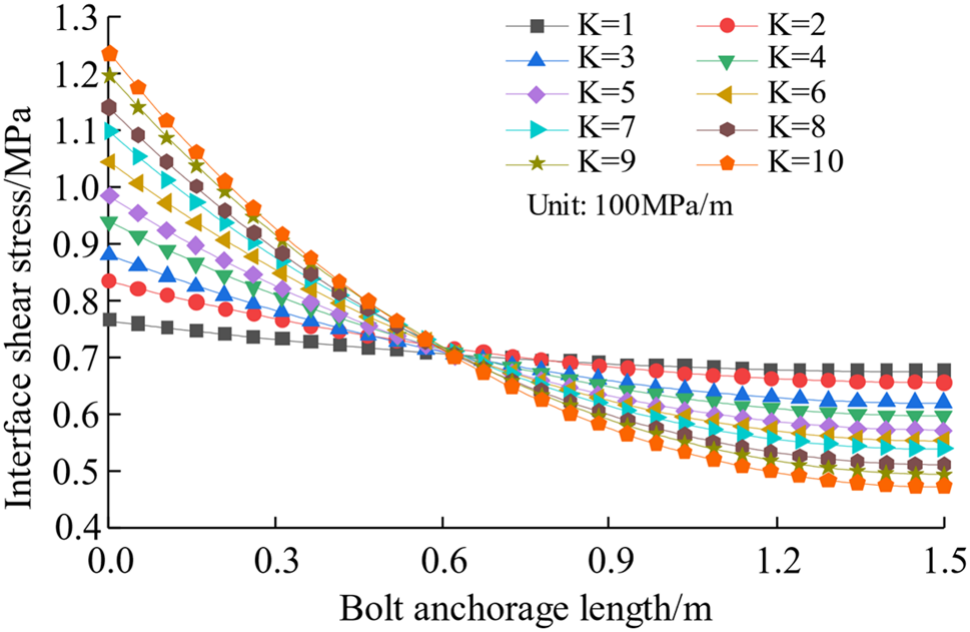
Influence of shear stiffness on interfacial shear stress distribution.

Taking the rock bolt preload force *P*_*a*_ = 100 KN and anchor length *L*_*a*_ = 1.5 m, in Fig. 7, we present the variation curve of interface shear stress with anchor length at different shear stiffness values. We can see from the graph that as the shear stiffness increases, that the higher shear stress at the origin of the anchor section decreases gradually towards the bottom. This implies that a larger shear stiffness of the interface leads to an uneven distribution of interface shear stress, and the more the shear stress concentration occurs at start of anchorage sections.

## Failure mechanism analysis of the anchoring system

### Field anchoring performance test of anchor bolt

So as to the maximal weight carrying capability of the bolted anchorage system and gather relevant parameters for subsequent research on the transfer of stress across the interface and the mechanisms of failure, we carried out the bolt pull-out tests in the roadways of 5302, 5308 and 5309 working faces of Changping Mine. In the test, the roof and both gang anchors were made of *φ*22 mm × 2400 mm left-handed rebar anchor bolt, the type of anchor cable for the roof of the roadway is SKP21.8-1/1720 × 7300, and the type of anchor cable for the two helpers of the roadway is SKP21.8-1/1720 × 5300 with a drilling diameter of 30 mm. Table 1 shows the field pullout test results for the Changping coal mine roadway.

**Table 1:** Data of anchor pull-out test results in Changping mine.

Test roadway	Support type	Support location	Bolt (cable) length (m)	Anchorage section length (mm)	Ultimate pull-out force (kN)	Displacement (mm)	Unit anchoring force (kN mm^−1^)	Failure forms
53,021	Bolt	Roof	2.4	300	> 200	–	–	Unpulled
	Bolt	Rib	2.4	290	165	9.87	0.57	Unpulled
	Bolt	Rib	2.4	295	154	8.41	0.49	Unpulled
	Cable	Roof	7.3	265	106	12.45	0.40	Failure
	Cable	Rib	5.3	280	96	9.79	0.34	Failure
	Cable	Rib	5.3	270	90	13.56	0.33	Failure
53,081	Bolt	Roof	2.4	300	> 200	–	–	Bolt broken
	Bolt	Rib	2.4	310	175	11.53	0.56	Unpulled
	Bolt	Rib	2.4	285	150	10.44	0.53	Unpulled
	Cable	Roof	7.3	285	100	8.73	0.35	Failure
	Cable	Rib	5.3	320	85	9.06	0.27	Failure
	Cable	Rib	5.3	280	93	10.64	0.33	Failure
53,093	Bolt	Roof	2.4	300	> 200	–	–	Unpulled
	Bolt	Rib	2.4	320	160	9.01	0.50	Unpulled
	Bolt	Rib	2.4	310	165	10.30	0.53	Unpulled
	Cable	Roof	7.3	270	110	8.41	0.41	Failure
	Cable	Rib	5.3	260	90	9.54	0.35	Failure
	Cable	Rib	5.3	255	87	12.87	0.34	Failure
Average				288.3	121.13	10.31	0.42	

The test showed that the main type of anchorage failure in mining roadways is the interfacial failure between the anchoring binder and the ambient rock mass, followed by interfacial failure. Limit pull-out force of bolts (cable) is between 85 and 175 kN, with an average value of 121.13 kN. For the max pulling force, the axial displacement of the stone bolts ranged from 8.41 to 13.56 mm, with an average displacement of 10.31 mm. The unit anchorage force is between 0.2 and 0.57 kN/mm, with an annual average weight of 0.42 kN/mm.

### Inversion of interface parameters based on field tests

Considering that the surface of the roadway is not flat during the field test, the surface rock will be gradually compacted during the process of applying preload to the thumbtack. Basis our analysis, the displacement corresponding to the maximum pullout load obtained from the test comprises two components: the displacement associated with the failure of the gradual of the secondary interface, and the displacement resulting from the compression of the anchor clamped perimeter rock. Therefore, the initial displacement of the damage should be smaller than the displacement corresponding to the peak pullout load obtained from the test when the numerical calculation is carried out, and *δ*_0_ takes to be 6.40 mm.

Only analyze the tests in which anchorage failure occurred during anchorage performance testing. Calculate the ultimate average shear stress and interface stiffness of the second interface according to Eqs. (10) and (11), respectively.

10 $$ \tau_{2,\max } = P_{\max } /\pi DL_{a} $$

11 $$ K = \tau_{2,\max } /\delta_{0} $$

where *τ*_*2,max*_ is the ultimate average the shearing stress at the sub-interface; *P*_*max*_ is the maximum pullout load of the screw.

Table 2 shows the calculation results. From the results, we have access to the geology of the mining tunnels in the Changping Mine. The ultimate shear stresses at the secondary surfaces of the anchoring system ranged from 2.84 to 6.04 MPa, with the lower limit value falling within this range, with an average of 4.46 MPa; the interface stiffness is between 0.44 and 0.94 MPa/mm, with an average value of 0.70 MPa/mm.

**Table 2:** Results of theoretical inversion interface parameters.

Test no	Length of anchored section (mm)	Ultimate pull-out force (KN)	Displacement (mm)	Ultimate shear stress (MPa)	Interface shear stiffness (MPa mm^−1^)
1	255	87	12.87	3.62	0.57
2	260	90	9.54	3.67	0.57
3	265	106	12.45	4.25	0.66
4	270	110	8.41	4.32	0.68
5	270	90	13.56	3.54	0.55
6	280	96	9.79	3.64	0.57
7	280	93	10.64	3.53	0.55
8	285	100	8.73	3.72	0.58
9	285	150	10.44	5.59	0.87
10	290	165	9.87	6.04	0.94
11	295	145	8.41	5.22	0.82
12	310	175	11.53	5.99	0.94
13	310	165	10.30	5.65	0.88
14	320	160	9.01	5.31	0.83
15	320	85	9.06	2.84	0.45
Average	288.33	121.13	10.31	4.46	0.70

### Numerical simulation analysis of failure mechanism of the anchorage system

#### Numerical calculation model

**Figure 8:**
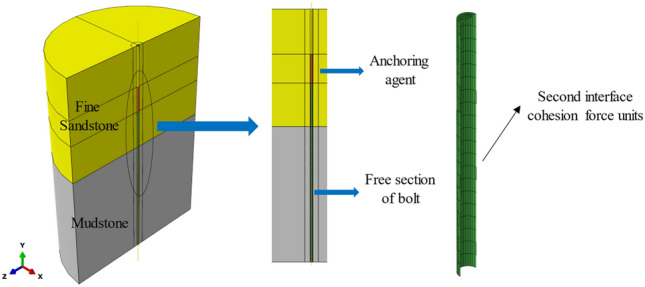
Finite element geometry model.

A three-dimensional anchoring system model was established through Abaqus numerical simulation software, as shown in Fig. 8. The model is a cylinder with a 4 m diameter and 3.3 m height, the corkscrew specification is *φ*22 × 2400 mm, and the rock bolt anchorage length is 1 m. Constrain the displacement of the model's sides and top, the bottom interface of the model is free, and simulate the surface protection action of the pallet by controlling the shaft displacement of the external surfaces the encompassing rock having a radius of 75 mm near the borehole. Insertion of zero-thickness cohesion cells at the anchor and perimeter rock interface to simulate sliding phenomena at the surface. The failure criterion of the cohesion force units obeys the *BK* criterion. The loading method was displacement-controlled linear loading with a displacement velocity of 20 mm/s imposed on the free edge of the rock bolt.

Both the rock bolt and resin anchorage adopt the linear-elastic model. The surrounding rock obeys the Mohr–Coulomb composition model. The rock bolt, resin anchorant, and surrounding rock use solid units, with the parameters listed in Table 3.

**Table 3:** Material parameters of finite element model.

Material type	Elastic modulus (MPa)	Poisson’s ratio	Angle of internal friction (°)	Swell angle (°)	Cohesion (MPa)
Rock bolt	200,000	0.30	–	–	–
Resin anchor agent	16,000	0.30	–	–	–
Mudstone	2890	0.29	36	18	4.33
Fine sandstone	5750	0.22	46	25	7.50

#### Results and analysis of numerical simulations

**Figure 9:**
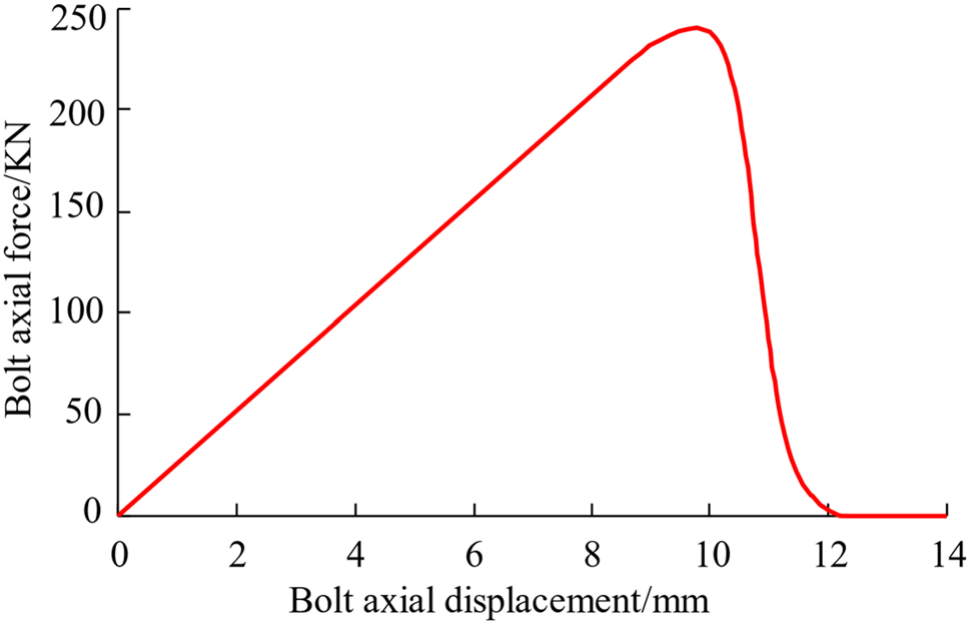
Curve of bolt body axial force versus bolt axial displacement.

Figure 9 shows the curve of the relationship of axial force on the body of the rock bolt to the axial displacement of the free end obtained by simulating the entire fracture phase of the stone bolt during its operation, including the linear phase of elasticity and the phase of relaxation after reaching the peak value. During the loading process, the largest axial force on rock bolts is 239.70 kN, with an end displacement of 9.8 mm. When axial load is reduced to zero, the terminal displacement is approximately 12.6 mm. The left-handed rebar anchor bolt without transverse reinforcement used for roof support in Changping Mine has a yield strength of 500 MPa, a yield force of 190 kN, and an ultimate tensile stress of 630 MPa or a breaking force of 240 kN. The numerical simulation results show that the preload-displacement curve enters the descending section when the bolt reaches the breaking force. In order to compare the state before and after the peak of thrust force, the value of the thrust force in the rising phase before the peak is labelled as "+" and the value of the thrust force in the falling phase after the peak is labelled as "−".

**Figure 10:**
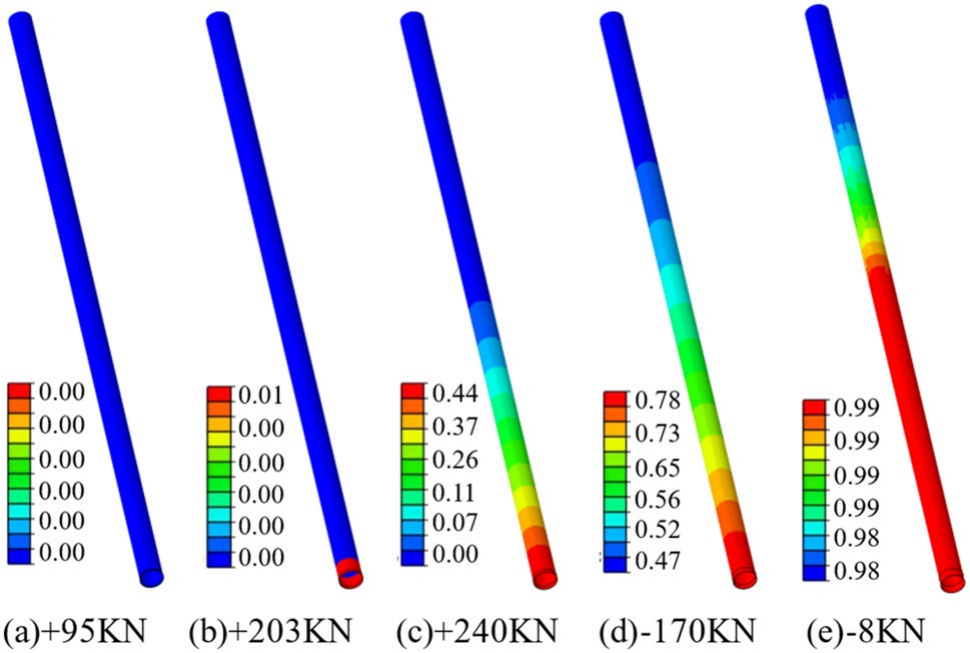
Cohesion force units damage factor cloud diagram.

**Figure 11:**
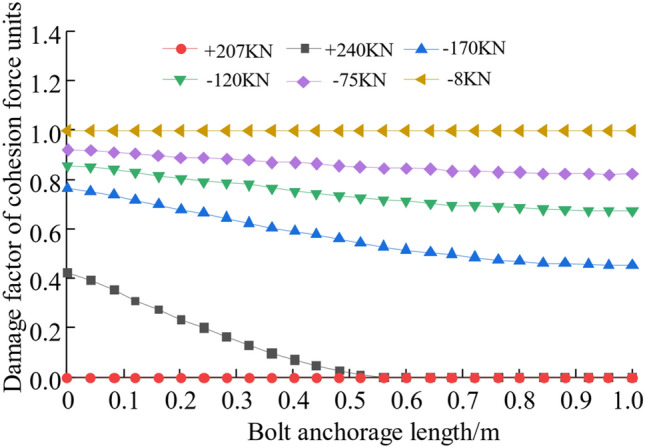
Damage factor curve of cohesion force units.

The interface failure can be determined by assessing the level of damage inflicted upon the cohesion force units, which are inserted at the junction between the anchoring agent and the interface of the perimeter rock. The degree of corruption of the cohesive unit is expressed by the corruption factor *D*. When the damage factor is 0, it indicates that the unit has not been damaged. When it is 1, it means that the unit has been completely damaged. Between 0 and 1, the cohesion force units are in the stage of stiffness degradation. The damage factor cloud chart of the cohesion force units for the second interface under various axial forces is shown in Fig. 9, and the damage factor curve of the cohesion force units for the second interface is shown in Fig. 10. Combined with Figs. 10 and 11, that when the axial force is 95 kN before the peak value, the cohesion force units of the second interface has not been damaged; when the axial force is 207 kN before the peak value, the cohesive unit at the start of the second interface anchorage section starts to enter the stiffness degradation phase; when the axial force reaches the peak value of 240 kN, the cohesion force units at about 0.6 m from the beginning of the anchor section all enter the damaged state, and the cohesion force units at the beginning of the anchor section has the largest damage degree, which is 0.44; when the axial force is 170 kN after the peak value, the damage factor of the cohesion force units for the second interface is between 0.47 and 0.78; when the axial force is 8 kN after the peak value, the damage factor of the cohesion force units for the second interface is close to 1, the second interface completely fails, and the anchor solid is completely pulled out.

**Figure 12:**
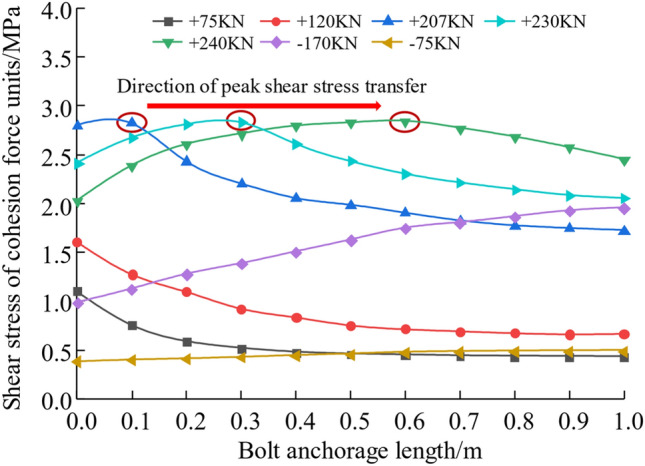
Cohesion force units shear stress distribution curve.

The extracted cohesion force units shear stresses at different stages are shown in Fig. 12. From the figure, it can be seen that in the absence of any damage to the anchor interface, the shear stress decreases gradually with the direction of the anchor length, and the shear stress is concentrated within one-third of the anchor length at the beginning of the anchor. With the damage of the cohesion force units of the anchorage interface to the stage of incomplete damage, the shear stress of the cohesion force units tends to increase first and then decrease. The largest shear stresses occur at locations where there is no interfacial damage, and as the interfacial damage moves inwards towards the anchorage end, the maximum shear stresses at the interface also move inwards. When the peak anchor axial force occurs after the stage of complete interface damage, at this time, the anchorage section is debonding but still can provide frictional resistance.

## Design of reasonable anchor length for anchor rods

### Anchor critical anchorage determination

According to Eq. (9), the hyperbolic function tanh (*βL*_*a*_) can only be infinitely close to 1. When *βL*_*a*_ = 3, tanh(*βL*_*a*_)≈1, and the bearing capacity can reach the maximum. At this stage, the depth of the anchoring section has no effect on the magnitude of the maximum bearing capacity. Continuing to extend the length by increasing the length of the anchorage section will not continue to increase the maximum load capacity. The ultimate ball bearing capacity *P*_*e,max*_, and the corresponding critical length of anchorage *L*_*b*_ of the bolt are expressed as:

12 $$ P_{e,\max } = \frac{\pi D[\tau ]}{\beta } $$

13 $$ L_{b} = 3/\beta = 1.5\sqrt {ED/K} $$

It can be observed from Formula (13) that there exists a negative correlation between the shear stiffness of the interface and the critical bolt length. On the contrary, the equivalent modulus of elasticity and bolt diameter are positively correlated with the length of the critical anchorage.

Assuming that the ratio of the bolt bearing volume before reaching the critical anchorage length to the bolt's ultimate load capacity at the candidate bolt length is λ.

14 $$ \lambda = \frac{{P_{e} }}{{P_{e,\max } }} = \frac{{\tanh (\beta L_{a} )}}{{\tanh (\beta L_{b} )}} $$

Table 4 shows the ratio of the anchorage length *L*_*a*_ to the bolt critical length *L*_*b*_ for different values of *λ.* According to the data in Table 4, shortening the anchorage length within the critical range of the corky can effectively maintain the utilization speed of the bolt without compromising the anchorage capacity. Therefore, the actual anchorage length can take 0.5*L*_*b*_. After reaching the critically anchored depth, a further increase in the anchor depth does not significantly increase the ultimate load carrying capacity at the bolt.

**Table 4:** Relationship of actual and critical anchorage length at different values of λ.

λ	0.5	0.6	0.7	0.8	0.8
*L*_*a*_/*L*_*b*_	0.18	0.23	0.29	0.37	0.5

### Reasonable anchorage length design process

1. *Safety aspects* The reasonable anchorage length of bolts should be designed with safety considerations in mind. That is, the design load *P*_*d*_ of the bolt should be lower than the breaking load *P*_*br*_ (*α*_1_*P*_*d*_ ≤ *P*_*br*_). And the shear stress at the beginning of the anchored segment should be lower than the shear strength of the anchored rock-envelope interface [τ] without slip debonding failure (*α*_2_*τ*_max_ ≤ [*τ*], *α*_1_, *α*_2_ is the safety factor).
2. *Economic aspects* The anchor design length *L*_*a*_ is as much lower than the required anchorage length *L*_*b*_ under the premise that the bolt can provide sufficient pullout force. *L*_*c*_ ≤ *α*_3_*L*_*b*_, *α*_3_ < 1.
3. The bolt design load is as close as possible to the elastic limit load *Pe* for the specified surrounding rock conditions, thus giving the anchor capacity of the enclosing rock full play.

**Figure 13:**
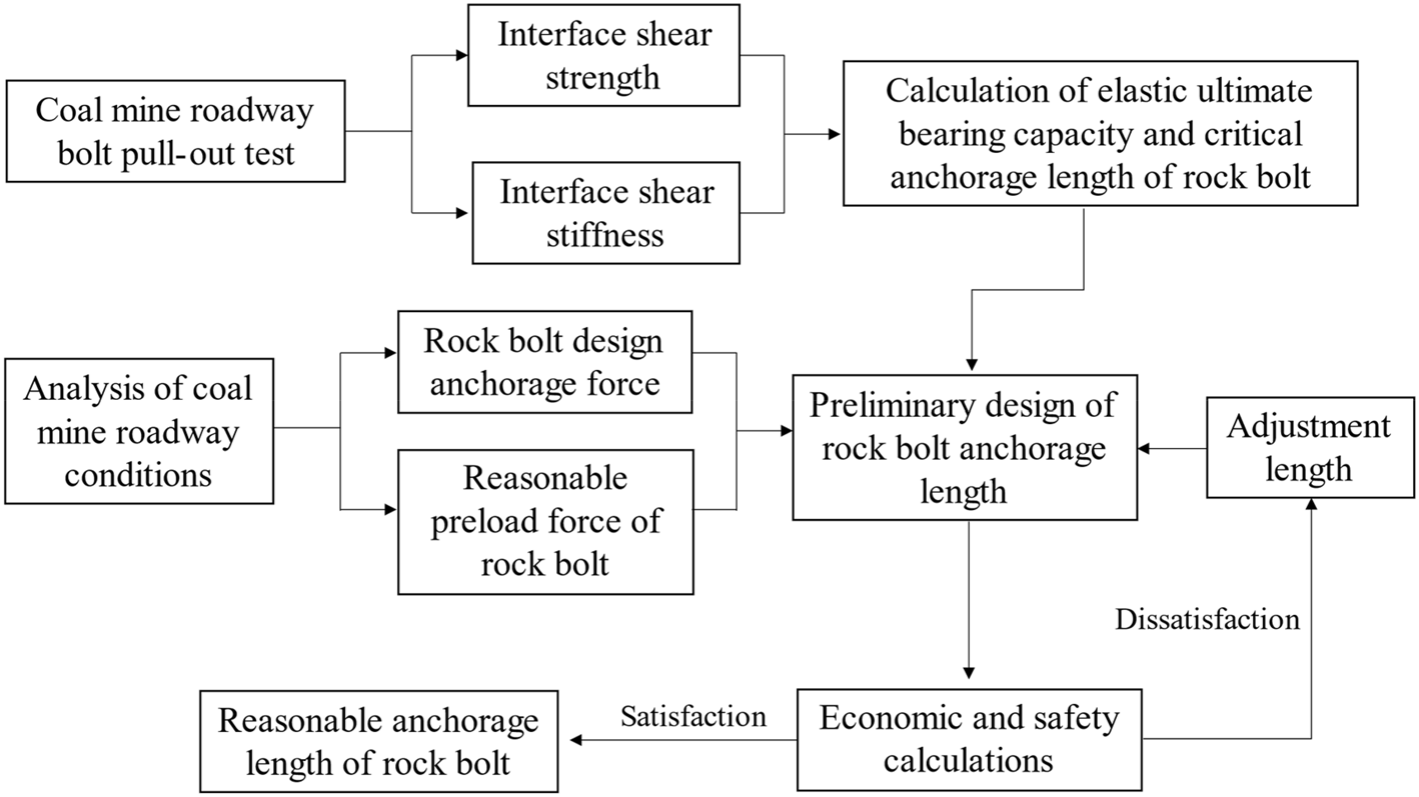
Design process of reasonable anchorage length of the bolt.

A comprehensive analysis of the above aspects, including determination of appropriate anchor lengths for the bolts, is illustrated in Fig. 13.

### Engineering application

Based on the bolt pulling test data of 5302, 5308, and 5309 working-face of Changping mine, take [*τ*] = 4.46 MPa and *K* = 700 MPa/m, and other parameters reference Table 3.

According to Eqs. (12) and (13), the rock bolt's ultimate bearing capacity and critical anchorage length are *P*_*e,max*_ = 467.05 KN, and *L*_*b*_ = 3.33 m, respectively. Assuming that the anchoring capacity is fully utilised in the envelope rock. In that case, the current mining rock bolts will have safety risks, and the length of mining rock bolts generally does not exceed 3 m, so the rock bolt anchorage length needs to be optimized.

For example, the MSGLW-500/22 × 2400 yield load is 190KN, and the breaking load is 240 KN. Taking the safety factor *α*_1_ = 1.5–2, the design load *P*_*d*_ = 120–160 KN. Comprehensive analysis shows that the surrounding rock's anchor ability and the anchor body's performance can be optimized when the design load is *P*_*d*_ = 160 kN and the anchorage length is 1.67 m. The maximum stress at the interface between the anchor and the rock is 1.04 MPa, which is less than the interface shear strength and meets the safety requirement.

The comprehensive analysis concluded that the reasonable anchoring length of rock anchors designed according to the slip and debonding fault of the interface between the anchoring agent and the surrounding rock is consistent with the current value of the actual anchoring length of the coal mine project, which verifies the basic principle of determining the reasonable anchoring length of the anchors and the feasibility of the design process.

## Conclusion

1. The analytical formula of stress distribution in the anchorage section is derived by establishing the anchorage system model. Factors affecting the damage of anchor interface were analysed: extreme bearing load of anchor is mainly related to the interface shear strength as well as shear stiffness, increasing anchor length appropriately can improve the interface damage threshold, and reducing interface shear stiffness can avoid the stress concentration phenomenon.
2. Field tests of bolt pull-outs indicate that anchor system failure occurs at the second interface. Bolts with [*τ*] or *K* obtained by the bolt pull-out test are the key parameters for the design of reasonable anchorage length.
3. The damage evolution law of the anchorage system was obtained through numerical simulation. As the anchorage unit initially enters a damaged state, there is a decrease in its shear stress-carrying capacity. Peak shear stress transmitted from the start of the anchored segment to the bottom of the anchored segment. That is, the interface failure process gradually damages from the beginning of the anchorage until the whole anchorage section.
4. Based on the failure mechanism of bolting systems, we present a approach for determining the appropriate length of anchorage for non-full-length resin-bonded anchors in coal mine roadways. The method can effectively utilise the surrounding rock anchorage capacity while ensuring safety and cost-effectiveness by making full use of the performance of the anchor material.

## Data Availability

This publication contains all the data produced or modelled in this study.
